# Chromosomal radiosensitivity and acute radiation side effects after radiotherapy in tumour patients - a follow-up study

**DOI:** 10.1186/1748-717X-6-32

**Published:** 2011-04-07

**Authors:** Reinhard Huber, Herbert Braselmann, Hans Geinitz, Irene Jaehnert, Adolf Baumgartner, Reinhard Thamm, Markus Figel, Michael Molls, Horst Zitzelsberger

**Affiliations:** 1 Department of Radiation Cytogenetics, HelmholtzZentrum Muenchen - German Research Center for Environmental Health, Neuherberg, Germany; 2 Department of Radiation Oncology, Technische Universitaet Muenchen, Munich, Germany; 3 Personal Monitoring Service, HelmholtzZentrum Muenchen - German Research Center for Environmental Health, Munich, Germany

## Abstract

**Background:**

Radiotherapists are highly interested in optimizing doses especially for patients who tend to suffer from side effects of radiotherapy (RT). It seems to be helpful to identify radiosensitive individuals before RT.

Thus we examined aberrations in FISH painted chromosomes in *in vitro *irradiated blood samples of a group of patients suffering from breast cancer. In parallel, a follow-up of side effects in these patients was registered and compared to detected chromosome aberrations.

**Methods:**

Blood samples (taken before radiotherapy) were irradiated *in vitro *with 3 Gy X-rays and analysed by FISH-painting to obtain aberration frequencies of first cycle metaphases for each patient. Aberration frequencies were analysed statistically to identify individuals with an elevated or reduced radiation response. Clinical data of patients have been recorded in parallel to gain knowledge on acute side effects of radiotherapy.

**Results:**

Eight patients with a significantly elevated or reduced aberration yield were identified by use of a t-test criterion. A comparison with clinical side effects revealed that among patients with elevated aberration yields one exhibited a higher degree of acute toxicity and two patients a premature onset of skin reaction already after a cumulative dose of only 10 Gy. A significant relationship existed between translocations *in vitro *and the time dependent occurrence of side effects of the skin during the therapy period.

**Conclusions:**

The results suggest that translocations can be used as a test to identify individuals with a potentially elevated radiosensitivity.

## Background

So far, a central problem for radiotherapy is the necessity to avoid severe side effects to normal tissues.

Thus, the irradiation dose which can be normally applied is limited by radiation response of the most radiosensitive tumour patients. As a consequence of such a protocol, lower than optimal irradiation doses will be applied to many patients. The lower doses affect the chance to achieve a better local tumour control.

Suitable cytogenetic tests might provide a crucial basis for an individualized radiotherapy. As a result, enhanced cytogenetic effects in single individuals might refer to enhanced tissue effects.

The dose response to radiotherapy might simply be analysed in peripheral blood cells before the beginning of radiotherapy.

## Introduction

Side effects in the normal tissues pose strong limitations for efficient radiotherapy of malignant cancers [1]. Severe normal tissue reactions affect mostly radiosensitive individuals who account for about 5% of all patients [2]. Therefore, radiation doses in treatment of cancer are generally restricted in order to minimize the incidence of such severe side effects which conversely imposes cure limitations for cancer treatment. For radiation biology it is therefore a major goal to identify predictors for increased radiosensitivity before treatment in order to allow an individualization of radiotherapy [3], thus optimizing tumour control rates and minimizing severe radiotherapy effects.

In addition, cancer risk for the rise of secondary tumours might increase in radiosensitive individuals [4].

There are many biological endpoints which could be used as a molecular predictor of radiosensitivity. Chromosomal aberration frequency is regarded as a good indicator because chromosomal aberrations are usually related to an altered DNA repair function which is in turn linked to cellular radiosensitivity for which dysfunction of many repair proteins have been demonstrated [2]. De Ruyck et al. [5] reported an enhanced chromosomal radiosensitivity detected by G2 assay as a marker of genetic predisposition to head and neck cancer. Borgmann et al. [6] found an important heredital impact with regard to radiation response detected by different cytogenetic assays (G0 test, G2 test) in lymphocytes of a collective of twins. Increased radiosensitivity of chromosomes in peripheral lymphocytes from cancer susceptibility syndrome patients, measured by chromosome breaks, was detected by Distel et al. [7]. The cited effect seems in several patients to be due to genetic instability [8]. Correlations between chromosomal aberration frequencies (chromosome aberrations or micronucleus frequency) and acute tissue effects after radiotherapy were reported by different authors [1,8,9]. In another study investigating radiation-induced DNA primary damage and repair kinetic, by use of the COMET assay [10], DNA effects were correlated with acute tissue effects, whilst in a study of Popanda et al. [11] a correlation of acute side effects with DNA degradation using the COMET assay could not be established. For late tissue effects correlations with genomic alterations detected by different assays have also been reported [1,8,12-15], however, the influence of other factors could not be excluded before such late tissue effects appeared in these clinical studies. Although the micronucleus test is often regarded as highly suited in clinical applications because of its simplicity, reproducibility and promptness [2] it turned out in several studies [16-18] that the analysis of chromosomal aberrations in FISH(fluorescence *in-situ *hybridisation)-painted metaphases is a very sensitive marker correlated to tissue reactions like acute skin effects or lesions. This leads us to investigate whether chromosomal aberrations can be used as a predictive marker to detect individuals showing a diverging radiosensitivity. To make a FISH-based assay for the detection of chromosomal aberrations more attractive for clinical applications we have combined the FISH procedure with an automated scoring of FISH-painted chromosome aberrations. This assay provides even hardly detectable cytogenetic endpoints like translocations and colour junctions.

In the present study, chromosomal radiosensitivity has been investigated in 47 breast tumour patients after *in vitro *irradiation of blood samples. FISH-painting has been applied to detect aberrations on chromosome 1, 4 and 12 (partial genome analysis, [19]), whilst acute tissue effects have been prospectively monitored during radiotherapy of these patients.

## Material and methods

### Patients

The collective was selected from patients of the radiological clinic that had to be subdued to radiotherapy under similar schemes of radiotherapy, without application of additional chemotherapeutic drugs. These conditions delivered 47 patients examined in the sequence of their reception in the clinic, who received exclusively radiotherapy due to a malignant breast tumour after surgical lumpectomy. Individual blood sampling was done within a follow-up period of six weeks.

The study was approved by the ethics committee of the University hospital Rechts der Isar of the Technical University Munich and done in accordance with the revised Declaration of Helsinki.

### Radiotherapy techniques

All patients were treated with 6 - 15 MeV photons from a linear accelerator. Dose per fraction was 1.8 - 2.0 Gy applied five times per week. Patients who received adjuvant radiotherapy after breast conserving surgery for breast cancer, were treated via tangential fields to the ipsilateral breast. After a cumulative dose of 50 Gy an electron boost with 10 -16 Gy to the former tumour region was performed.

### Side effects of radiotherapy

Clinical side effects of radiotherapy were evaluated weekly during radiotherapy. Scoring was carried out according to the Common Toxicity Criteria (NCI-CTC scale; scale digits 0, 1, 2, 3, 4). Mainly skin effects have been identified as side reactions of radiotherapy.

### Irradiation procedure *in vitro *and lymphocyte cultures

Whole blood samples (4ml fractionated in 2× 2 ml syringes) were irradiated *in vitro *with 3 Gy of 220 kV X-rays (15 mA, 0.5 mm Cu and 4.05 mm Al filters, dose rate 0.5 Gy min^-1^) at 37°C. Immediately after irradiation, whole blood cultures were initiated according to our published protocol [20]. Moreover, BrdU (final concentration 9.6 x10^-6 ^μg ml^-1^) was added to the cultures for identification of 1^st ^cell cycle chromosomes. Cultures were incubated at 37°C for 48 h involving a colcemid treatment (0.1 mg ml^-1^) for the final three hours.

Chromosome preparation was performed according to standard procedures with slight modifications of our published protocol [19]. Microscopic slides were stored in a nitrogen atmosphere at -20°C until use.

### FISH (fluorescence *in-situ *hybridisation)

For a homogeneous staining of three chromosome pairs, FISH with painting probes for chromosomes 1, 4, and 12 directly labelled with FITC (probe set ID005, Chrombios, Raubling, Germany), together with a pancentromeric DNA probe was applied according to manufacturer's manual. Counterstaining was performed with propidium iodide (PI, 1 μg ml^-1^) in antifade solution. Before hybridisation, slides were treated with thiocyanate for 10 min at 90°C instead of pre-treatment with pepsine [21]. For a discrimination between first and second cycle metaphases (harlequin staining), prior to painting, slides were treated with bisbenzimide (H33258, Serva, Heidelberg, Germany) and UV light as described by our published protocol [22].

### Chromosome analyses

Metaphase finding and image capturing was performed on a Metafer2 scanning system (Metasystems, Altlussheim, Germany) with a Zeiss Axioplan2 MOT microscope as described earlier [19]. Aberration analysis was carried out interactively on three-colour metaphase gallery images or on full screen images, both providing three colour channels on the display for the presentation of FISH painted chromosomes, of counterstained chromosomes, and of centromeric signals, using the PAINT nomenclature system [23] to describe the observed painting patterns. For the subsequent statistical analysis, painted chromosomes bearing one centromere with a colour junction were registered as t(Ab) or t(Ba), respectively, painted chromosomes with two centromeres and a colour junction as dicentrics. Painted chromosomes exhibiting an insertion, ace(b), and other aberration types, were registered but not subdued to statistical analysis.

Chromosome pairs 1, 4, and 12 appeared in green (FITC), the centromeres were stained in blue (AMCA), counterstaining of the complete metaphases appeared in red (PI). Due to preceding harlequin staining, chromosomes in first cycle metaphases have a homogeneous appearance, those in second cycle metaphases exhibit differential staining of sister chromatids. The latter were excluded from chromosome analysis.

A mean of 140 *in vitro *irradiated lymphocytes (variation 50 - 467) per patient was analysed. We protocoled all types of structural aberrations in painted chromosomes as follows: all types of symmetrical translocations, dicentrics, chromatid type aberrations, excess acentrics, the numbers of metaphases with/without structural aberrations, and colour junctions.

### Statistical methods

For statistical analysis of the degree of skin side reaction the maximum achieved scale digit during the follow-up period was scored. The homogeneity of chromosome aberration frequencies among the patient samples was examined by a χ^2 ^test. Correlations were analysed by Spearman's rank correlation test. Outlying frequencies were identified by a single classification t-test with p < 0.05 as criterion.

## Results

47 patients have been investigated for clinical side reactions and for *in vitro *response of peripheral lymphocytes to 3 Gy X-rays irradiation.

### Evaluation of clinical data

Skin reactions (NCI-CTC grading, common toxicity criteria of the US National Cancer Institute) during and after radiotherapy have been classified according to the following scale: grade 0: no skin reaction, grade 1: small erythema, depilation, dry dandruff, reduced perspiration; grade 2: moderate erythema, epitheliolysis <50% of radiation field, moderate edema; grade 3: large erythema, epitheliolysis >50% of radiation field, strong edema; grade 4: deep ulcer, haemorrhage or necrosis. 4 of 47 patients showed grade 0, 30 patients grade 1, 12 patients grade 2, and 1 patient grade 3.

As an additional grouping patients were classified according to the time-dependent occurrence of skin reactions in the order "early reaction" if it occurred after an accumulated dose of 10 Gy, as "in between reaction", if it occurred after 30 Gy accumulated dose, as "late reaction", if it occurred at the end of radiotherapy, and as "no reaction". 4 of 47 patients showed no reactions, 13 patients late reactions, 23 patients in between reactions, and 7 patients early reactions (individual data not shown, total data presented in "Additional file 1 Table S1".

### Evaluation of chromosome aberrations

FISH painting was performed on *in vitro *irradiated metaphase preparations which were further subdued to aberration analysis using the semi-automated Metafer2 system (Metasystems GmbH, Altlussheim, Germany). The following classifications of cytogenetic effects have been used for statistical treatment:

(i) all metaphases containing structural aberrations, (ii) translocations of the t(Ab) as well as t(Ba) types, (iii) dicentrics (dic), (iv) colour junctions (cj). This classification enables the detection of radiation-induced chromosome aberrations in total and subclassification into different aberration types.

**Figure 1:**
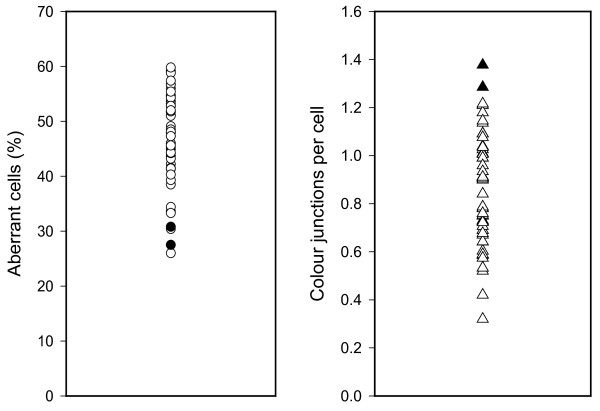
**Distribution of aberrant cells and of colour junctions in *in vitro *irradiated lymphocytes of 47 patients**. Symbols represent individual frequency of the respective cytogenetic endpoint. Filled symbols represent cases with significantly increased or decreased frequency. Exposure 3 Gy.

**Figure 2:**
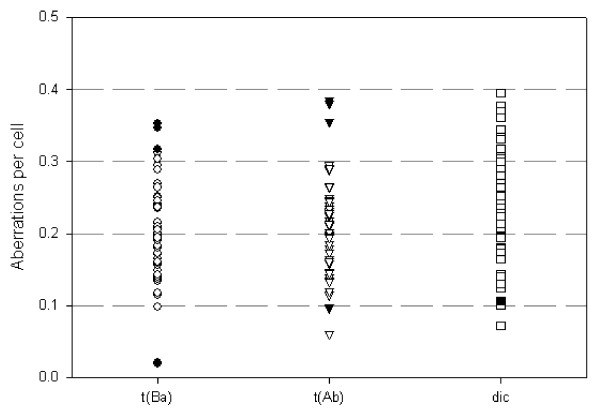
**Distribution of translocation types t(Ba), t(Ab), and of dicentrics (dic) in *in vitro *irradiated lymphocytes of 47 patients**. Symbols represent respective individual frequency of respective aberration type. Filled symbols represent cases with significantly increased or decreased frequency. Exposure 3 Gy.

A total of 6829 metaphases were analysed and individual chromosome aberration yields were compared for 47 patients. Aberration yields are shown for the respective cytogenetic effect in Figures 1 and 2.

Numerical data of aberrations are shown in table "Additional file 2 Table S2".

Statistical analyses revealed that for all patients investigated different aberration types are correlated to each other. This can be demonstrated for the yields of t(Ab) corresponding t(Ba) (p < 0.0001), for t(Ba) and corresponding dicentric yields (p < 0.0037) and for t(Ab) and the corresponding dicentric yields (p < 0.0072). Moreover, a significant overdispersion, i.e. a non-homogeneous distribution among patient samples (p < 0.0001) was found for all cytogenetic effects (t(Ba), t(Ab), dicentrics, colour junctions, cells containing aberrations). The median frequencies were 0.20 per cell for t(Ba), 0.21 for t(Ab) and 0.26 for dicentrics.

The chromosome analysis revealed several patients that show a conspicuously higher or lower aberration yield, respectively. A single classification test was further used to identify single patients with a significant deviation from the mean aberration frequencies. Results are summarised in Table 1 showing significantly raised aberration frequencies for patients 1 and 3 (t(Ba)/cell, t(Ab)/cell, colour junctions/cell), for patient 7 (t(Ba)/cell) and for patient 17 (t(Ab)/cell). Significantly reduced aberration frequencies are revealed for patient 30 (dicentrics/cell), for patient 36 (t(Ab)/cell, structural aberrations/aberrant cell, for patient 37 (t(Ba)/cell) and for patient 41 (structural aberrations/aberrant cell).

**Table 1:** Patients exhibiting a significant deviation from the mean at different aberration types (likelihood quotient test, p < 0.01)

patients	cytogenetic endpoint				
	**C_A _(%)**	**t(Ba)/cell**	**t(Ab)/cell**	**dic/cell**	**cj/cell**

significantly increased cytogenetic effects					

patient 1	52.7	***0.317***	***0.385***	0.336	***1.377***

patient 3	53.7	***0.347***	***0.355***	0.238	***1.285***

patient 7	54.7	***0.353***	0.264	0.259	1.209

patient 17	51.7	0.313	***0.381***	0.224	1.136

significantly reduced cytogenetic effects					

patient 30	49.1	0.171	0.239	***0.107***	0.585

patient 36	***30.8***	0.149	***0.097***	0.144	0.533

patient 37	26.0	***0.020***	0.060	0.140	0.320

patient 41	***27.5***	0.183	0.174	0.174	0.642

C_A_%: percentage of cells containing structural aberrations

*Italic*: significant difference to mean value of patients

### Correlation between chromosome aberrations and clinical side effects

**Figure 3:**
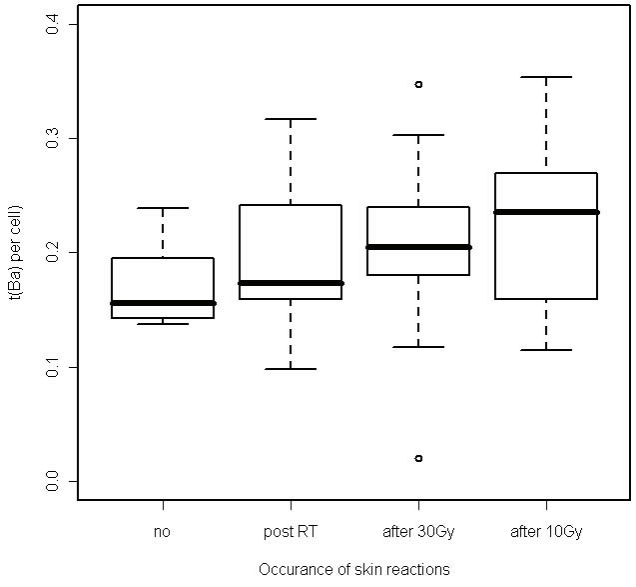
**Box plot analysis of t(Ba) frequencies in 4 patient groups ordered according to temporal occurence of any side effects of the skin during the period of radiation therapy**. Box area, 50% of data [lines in box denote medians; bars include at most 1.5 of interquartile distance, difference between first and third quartiles of data; circles indicate values out of the 1.5-fold box area (outliers)]. A significant correlation between the frequencies of t(Ba) from lymphocytes irradiated *in vitro *(3 Gy) and the time-dependent occurence of side effects is demonstrated.

A comparison of individual chromosome aberration data with clinical side reactions revealed that among patients with increased aberration yields (all of them treated with identical doses of 50 Gy and 10 Gy boost) patient 1 exhibited more severe side effects and patients 7 and 17 showed early reactions after 10 Gy. Patient 3 with an increased chromosomal sensitivity did not show an increased acute side reaction. Apart from individual chromosomal outliers a significant overall correlation was found between the frequencies of t(Ba) *in vitro *and the time-dependent occurence, i.e. latency of side effects of the skin (Spearman's rank correlation test, p = 0.014). The correlation is shown in Figure 3.

In practice, a discrimination of patients is done using cut-off levels. The median t(Ba) frequency in the group of patients showing a skin reaction already after 10 Gy (short latency) is 0.21 per cell. In the group of patients showing no skin reaction or not before 30 Gy (longer latency) the t(Ba) median is 0.17 per cell. Taking the mean 0.19 per cell as a cut-off, the low frequency group (<0.19, 19 patients) and high frequency group (> 0.19, 28 patients) are associated to the latency groups with a Fisher's exact test p-value of 0.015. With this cut-off 22 of the 30 short latency patients (73.3%) are correctly detected (sensitivity) and 11 of the 17 longer latency patients (64,7%) are correctly assigned (specifity). For the endpoints t(Ab), dic and cj no correlation with side effects or with latency was found (see test results in "Additional file 3 Table S3").

## Discussion

The aim of this study was to investigate the relationship of chromosomal radiosensitivity and acute clinical side effects in 47 breast cancer patients who underwent radiotherapy for tumour treatment. The extent of clinical side effects has been used as an indicator for the individual radiosensitivity of each patient. Such established relationships would be of clinical relevance because they could represent a predictive factor that is required for an individualisation of radiotherapy [2].

Greve at al. [24] reasoned that neither measurement of radiation-induced apoptotic and necrotic cell death is detectable in immortalised lymphoblastoid derivatives nor cell death in blood lymphocytes is suitable to unequivocally predict the individual clinical radiosensitivity of cancer patients.

Premature chromosome condensation (G2 test) reveals practically indistinguishable levels of chromosomal breaks in AT and normal lymphoblastoid cells or lymphocytes, though lymphocytes of AT patients reveal an increased radiosensitivity measured by PCC(premature chromosome condensation) chromosome breaks [25].

Based on the micronucleus assay in cytokinesis-blocked lymphocytes, Mozdarani et al. [26] found significant differences between a control group and groups of breast cancer or oesophageal cancer patients, respectively, after in vitro irradiation with 3 Gy; nevertheless, radiosensitive individuals could not be identified in this study.

Interindividual radiosensitivity in blood lymphocytes of 14 healthy donors could not be detected with the micronucleus assay, nor with the G2 assay. It could not be decided whether the detected variation of both cytogenetic effects was due to interindividual variation of radiosensitivity, or to intraindividual variation [27]. Hence it is promising to study chromosomal damage as a marker for cellular radiosensitivity because it is well established as a quantitative indicator for preceding radiation exposure [28-33]. We therefore have quantified chromosomal aberrations in blood samples from 47 tumour patients which have been irradiated with 3 Gy X-rays *in vitro*. The measured aberration frequencies showed for some patients significant deviations from the mean value for each aberration category (Figures 1 and 2). The presented approach is novel because in this study the use of an automated scoring system allowed an evaluation of 6829 metaphases which would facilitate to use this approach routinely in clinical testing. The validity of these scoring results is indicated by the highly significant correlations between each aberration categories.

The statistical analyses further revealed that four out of 47 patients exhibited a significantly elevated aberration frequency at least for one aberration category indicating an increased radiation response at the DNA repair level (Table 1). Interestingly, the dicentric frequencies were not significantly elevated in each of the four patients, but translocations showed a significant increase. Such discrepancies between translocation and dicentric yields after radiation exposure have already been described in several studies quantifying radiation-induced chromosome aberrations [32,34]. In view of the correlation, it means that translocations show a more extensive response to radiation compared to dicentrics.

So far, Keller et al. [17] reported that among other cytogenetic parameters, the parameter "percentage of dicentric chromosomes" could neither serve as meaningful nor as significant criteria, since it showed a strong interindividual variability, whereas translocations were suitable indicators for detecting differences in blood lymphocytes from patients and controls irradiated *in vitro *with two different doses.

On the other hand there was found an indication for a reduced radiation response since significantly reduced aberration frequencies at least for one aberration category have been detected in four patients (Table 1). Thus based on cytogenetic results one would expect four patients with an enhanced and four patients with a reduced radiosensitivity in our study. In order to validate this assumption, clinical phenotypes were also considered. The comparison with acute clinical side effects (mainly skin reactions) demonstrated that none of the patients exhibiting significantly reduced aberration yields suffered from abnormal tissue reactions during or after radiotherapy reflecting the initial finding of a reduced radiosensitivity. However, among the four patients with elevated aberration frequencies three patients showed either a more severe side reaction of radiotherapy (patient 1) or a premature side reaction already after 10 Gy of irradiation (patients 7 and 17). Although such a co-incidence could not be found for patient 3, these results let assume that a relationship between cellular radiosensitivity measured as chromosome aberration yield in peripheral lymphocytes and acute clinical side reactions exists. Anyway, it could be demonstrated with statistical significance that a chromosome aberration test investigating translocations by FISH is suitable to identify individuals with shortened response time of radiation-induced skin reactions.

So far, only few studies exist reporting on similar relationships between acute clinical reactions and metaphase chromosome radiosensitivity. Dunst et al. [12] demonstrated that nine out of 26 radiotherapy patients showing elevated chromosome break frequencies suffered from an increased acute skin damage. Compared to our patient cohort they investigated more different tumour types leading to higher heterogeneity after *in vitro *exposure with 0.7 and 2.0 Gy in the study group [12]. Similar results were reported by Popanda et al. [11] who detected 6 out of 113 radiotherapy patients with excessive acute skin reactions also showing significantly increased radiation-induced genomic changes detected by the COMET assay. However, a statistical correlation between genome alterations and acute side effects could not be demonstrated. Further studies reported an increased cellular radiosensitivity in radiotherapy patients using G0 and G2 assays [27,35]. However, these did not register clinical side effects which limits the impact of their results. On the other hand in a recent study, Slonina et al. [36] could not find elevated acute or late side effects in cervix carcinoma patients whose cultured keratinocytes and fibroblasts exhibited increased micronucleus frequencies. Moreover, it has been demonstrated in several *in vitro *studies that the G0 micronucleus assay in blood lymphocytes using 3 Gy *in vitro *exposure [37], using 3.5 Gy *in vitro *exposure [27], and blood lymphocyte G2 assay using 0.4 Gy *in vitro *exposure [27], have limited reproducibility due to extended intraindividual variability. Limitations of the G2 assay, e.g. from interindividual variation, were also reported in a compilation from data of different studies [38].

In conclusion, a comparison of our findings with several published data suggests that measuring chromosomal radiosensitivity on translocation level in blood lymphocytes can be proposed to be used as a predictive assay for detection of radiosensitive individuals which should be developed further. Data from larger cohorts are needed to assess whether a particular aberration type is most sensitive to detect increased radiosensitivity. It would be also of interest to monitor chromosome aberrations in blood lymphocytes *ex vivo *at different times during radiotherapy to evaluate whether the occurrence of acute clinical side effects is related to increased aberration frequencies in a timely manner in order to detect a potential timely correlation, which would correspond to our findings from lymphocytes exposed *in vitro*.

## Competing interests

The authors declare that they have no competing interests.

## Authors' contributions

RH has substantially contributed to acquisition and interpretation of data; he has been involved in drafting the manuscript and has contributed to the final version to be published. HB has made substantial contributions to the conception and design of the study, to analysis and interpretation of the study. He was responsible for the statistical treatment of data, kindly delivering the manuscript's Figures. He was involved in drafting the manuscript and revising it critically, and has given final approval of the version to be published. HG has made substantial contributions to conception and design of the study. As a clinical radiologist, he supervised the administration and delivery of patients' blood samples. He has been involved in revising the manuscript critically and has given final approval of the version to be published. IJ has made substantial contributions to acquisition of data, collecting blood samples, and lymphocyte culture procedures. She has been involved in revising the protocol critically. AB has made substantial contributions to acquisition of data, collecting blood samples, lymphocyte culture and FISH procedures. RT has made substantial contributions to conception and design of the study. As a clinical radiologist, he supervised the administration and delivery of patients' blood samples. MF has delivered dosimetry for *in vitro *irradiation experiments, and he provided practical advice for the handling of the irradiation device. MM has made substantial contributions to conception and design of the study. HZ has made substantial contributions to conception and design of the study, and the interpretation of data. He has been involved in drafting the manuscript and revising it critically. He has given final approval of the version to be published.

All authors read and approved the final manuscript.

## Supplementary Material

Additional file 1 **Radiotherapy's side effects of 47 tumour patients**. Side effects of radiotherapy in 47 tumour patients (highest degree and occurrence of skin reaction). Click here for file

Additional file 2 **Absolute numbers of cytogenetic effects in *in vitro *irradiated blood lymphocytes of 47 tumour patients**. Absolute numbers of different types of cytogenetic effects from *in vitro *irradiated (3 Gy) blood lymphocytes of 47 tumour patients. Click here for file

Additional file 3 **Correlation coefficients of different types of chromosome aberrations from *in vitro *irradiated lymphocytes**. Correlation coefficients of different types of chromosome aberrations from *in vitro *irradiated (3 Gy) lymphocytes compared to degree of side effects and to latency of side effects in 47 patients (p-values for Spearman's rank correlation test). Click here for file

## References

[B1] Barber JB Burrill W Spreadborough AR Levine E Warren C Kiltie AE Roberts SA Scott D Relationship between *in vitro *chromosomal radiosensitivity of peripheral blood lymphocytes and the expression of normal tissue damage following radiotherapy for breast cancer Radiother Oncol 2000 55 179 186 10.1016/S0167-8140(99)00158-9 10799730

[B2] Sprung CN Chao M Leong T McKay J Chromosomal radiosensitivity in two cell lineages derived from clinically radiosensitive cancer patients Clin Cancer Res 2005 11 6352 6358 10.1158/1078-0432.CCR-04-1931 16144940

[B3] Sprung CN Davey DS Withana NP Distel LV McKay MJ Telomere length in lymphoblast cell lines derived from clinically radiosensitive cancer patients Cancer Biol Ther 2008 638 644 18364569 10.4161/cbt.7.5.5762

[B4] Dyomina EA Ryabchenko NM Increased individual chromosomal radiosensitivity of human lymphocytes as a parameter of cancer risk Exp Oncol 2007 29 217 220 18004249

[B5] de Ruyck K de Gelder V van Eijkeren M Boterberg T De Neve W Vral A Thierens H Chromosomal radiosensitivity in head and neck cancer patients: evidence for genetic predisposition? Br J Cancer 2008 98 1723 1738 10.1038/sj.bjc.6604345 18414410 PMC2391130

[B6] Borgmann K Haeberle D Doerk T Busjahn A Stephan G Dikomey E Genetic determination of chromosomal radiosensitivities in G0- and G2-phase human lymphocytes Radiother Oncol 2007 83 196 202 10.1016/j.radonc.2007.04.010 17499867

[B7] Distel LV Neubauer S Keller U Sprung CN Sauer R Grabenbauer G Individual differences in chromosomal aberrations after *in vitro *irradiation of cells from healthy individuals, cancer and cancer susceptibility syndrome patients Radiother Oncol 2006 81 257 263 10.1016/j.radonc.2006.10.012 17113667

[B8] Keller U Grabenbauer G Kuechler A Sprung CN Mueller E Sauer R Distel L Cytogenetic instability in young patients with multiple primary cancers Cancer Genet Cytogenet 2005 157 25 32 10.1016/j.cancergencyto.2004.05.018 15676143

[B9] Jones LA Scott D Cowan R Roberts SA Abnormal radiosensitivity of lymphocytes from breast cancer patients with excessive normal tissue damage after radiotherapy: chromosome aberrations after low dose-rate irradiation Int J Radiat Biol 1995 67 519 528 10.1080/09553009514550631 7775827

[B10] Sterpone S Cornetta T Padua L Mastellone V Giammarino D Testa A Tirindelli D Cozzi R Donato V DNA repair capacity and acute radiotherapy adverse effects in Italian breast cancer patients Mutat Res 2010 684 43 48 10.1016/j.mrfmmm.2009.11.009 19962393

[B11] Popanda O Ebbeler R Twardella D Helmbold I Gotzes F Schmezer P Thielmann HW von Fournier D Haase W Sautter-Bihl ML Wenz F Bartsch H Chang-Claude J Radiation-induced DNA damage and repair in lymphocytes from breast cancer patients and their correlation with acute skin reactions to radiotherapy Int J Radiat Oncol Biol Phys 2003 55 1216 1225 10.1016/S0360-3016(02)04415-2 12654430

[B12] Dunst J Neubauer S Becker A Gebhart E Chromosomal *in vitro *radiosensitivity of lymphocytes in radiotherapy patients and AT-homozygotes Strahlenther Onkol 1998 174 510 516 10.1007/BF03038983 9810318

[B13] Borgmann K Roper B El-Awady R Brackrock S Bigalke M Dork T Alberti W Dikomey E Dahm-Daphi J Indicators of late normal tissue response after radiotherapy for head and neck cancer: fibroblasts, lymphocytes, genetics, DNA repair, and chromosome aberrations Radiother Oncol 2002 64 141 152 10.1016/S0167-8140(02)00167-6 12242123

[B14] Hoeller U Borgmann K Bonacker M Kuhlmey A Bajrovic A Jung H Alberti W Dikomey E Individual radiosensitivity measured with lymphocytes may be used to predict the risk of fibrosis after radiotherapy for breast cancer Radiother Oncol 2003 69 137 144 10.1016/j.radonc.2003.10.001 14643950

[B15] Ramsay J Birrell G Normal tissue radiosensitivity in breast cancer patients Int J Radiat Oncol Biol Phys 1995 31 339 344 10.1016/0360-3016(94)00478-4 7836087

[B16] Keller U Grabenbauer G Kuechler A Sauer R Distel L Technical report. Radiation sensitivity testing by fluorescence in-situ hybridisation: how many metaphases have to be analysed? Int J Radiat Biol 2004 80 615 620 10.1080/09553000410001724568 15370973

[B17] Keller U Kuechler A Liehr T Mueller E Grabenbauer G Sauer R Distel L Impact of various parameters in detecting chromosomal aberrations by FISH to describe radiosensitivity Strahlenther Onkol 2004 180 289 296 10.1007/s00066-004-1200-y 15127159

[B18] Tucker JD Sensitivity, specificity, and persistence of chromosome translocations for radiation biodosimetry Mil Med 2002 167 8 9 11873525

[B19] Huber R Kulka U Loerch T Braselmann H Engert D Figel M Bauchinger M Technical report: application of the Metafer2 fluorescence scanning system for the analysis of radiation-induced chromosome aberrations measured by FISH-chromosome painting Mutat Res 2001 492 51 57 11377243 10.1016/s1383-5718(01)00151-6

[B20] Huber R Braselmann H Kulka U Schumacher-Georgiadou V Bayerl A Molls M Bauchinger M Follow-up analysis of translocation and dicentric frequencies, measured by FISH-chromosome painting in breast cancer patients after partial-body radiotherapy with little bone marrow exposure Mutat Res 1999 446 103 109 10613190 10.1016/s1383-5718(99)00153-9

[B21] Mueller I Geinitz H Braselmann H Baumgartner A Fasan A Thamm R Molls M Meineke V Zitzelsberger H Time-course of radiation-induced chromosomal aberrations in tumor patients after radiotherapy Int J Radiat Oncol Biol Phys 2005 63 1214 1220 10.1016/j.ijrobp.2005.03.056 16253775

[B22] Kulka U Huber R Mueller P Knehr S Bauchinger M Combined FISH painting and harlequin staining for cell cycle-controlled chromosome analysis in human lymphocytes Int J Radiat Biol 1995 68 25 27 10.1080/09553009514550881 7629434

[B23] Tucker JD Morgan WF Awa AA Bauchinger M Blakey D Cornforth MN Littlefield LG Natarajan AT Shasserre C PAINT: a proposed nomenclature for structural aberrations detected by whole chromosome painting Mutat Res 1995 347 21 24 10.1016/0165-7992(95)90028-4 7596363

[B24] Greve B Dreffke K Rickinger A Koenemann S Fritz E Eckardt-Schupp F Amler S Sauerland C Braselmann H Sauter W Illig T Schmezer P Gomolka M Willich N Boelling T Multicentric investigation of ionising radiation-induced cell death as a predictive parameter of individual radiosensitivity Apoptosis 2009 14 226 235 10.1007/s10495-008-0294-6 19142732

[B25] Terzoudi GI Manola KN Pantelias GE Iliakis G Checkpoint abrogation in G2 compromises repair of chromosomal breaks in ataxia telangiectasia cells Cancer Res 2005 65 11292 11296 10.1158/0008-5472.CAN-05-2148 16357135

[B26] Mozdarani H Mansouri Z Haeri SA Cytogenetic radiosensitivity of G_0_-lymphocytes of breast and esophageal cancer patients as determined by micronucleus assay J Radiat Res (Tokyo) 2005 46 111 116 10.1269/jrr.46.111 15802866

[B27] Vral A Thierens H Baeyens A De Ridder L The micronucleus and G2-phase assays for human blood lymphocytes as biomarkers of individual sensitivity to ionizing radiation: limitations imposed by intraindividual variability Radiat Res 2002 157 472 477 10.1667/0033-7587(2002)157[0472:TMAGPA]2.0.CO;2 11893251

[B28] Schmid E Bauchinger M Bunde E Ferbert HF von Lieven H Comparison of the chromosome damage and its dose response after medical whole-body exposure to 60Co gamma-rays and irradiation of blood *in vitro* Int J Radiat Biol Relat Stud Phys Chem Med 1974 26 31 37 10.1080/09553007414550911 4547374

[B29] Evans HJ Buckton KE Hamilton GE Carothers A Radiation-induced chromosome aberrations in nuclear-dockyard workers Nature 1979 277 531 534 10.1038/277531a0 763341

[B30] Pantelias GE Iliakis GE Sambani CD Politis G Biological dosimetry of absorbed radiation by C-banding of interphase chromosomes in peripheral blood lymphocytes Int J Radiat Biol 1993 63 349 354 10.1080/09553009314550461 8095285

[B31] Bauchinger M Braselmann H Savage JR Natarajan AT Terzoudi GI Pantelias GE Darroudi F Figgitt M Griffin CS Knehr S Okladnikova ND Santos S Snigiryova G Collaborative exercise on the use of FISH chromosome painting for retrospective biodosimetry of Mayak nuclear-industrial personnel Int J Radiat Biol 2001 77 259 267 10.1080/09553000010018693 11258840

[B32] Rao BS Natarajan AT Retrospective biological dosimetry of absorbed radiation Radiat Prot Dosimetry 2001 95 17 23 11468799 10.1093/oxfordjournals.rpd.a006516

[B33] Montoro A Rodriguez P Almonacid M Villaescusa JI Verdú G Caballín MR Barrios L Barquinero JF Biological dosimetry in a group of radiologists by the analysis of dicentrics and translocations Radiat Res 2005 164 612 617 10.1667/RR3444.1 16238438

[B34] Bauchinger M Schmid E Braselmann H Time-course of translocation and dicentric frequencies in a radiation accident case Int J Radiat Biol 2001 77 553 557 10.1080/09553000010022382 11382333

[B35] Baeyens A Thierens H Claes K Poppe B Messiaen L De Ridder L Vral A Chromosomal radiosensitivity in breast cancer patients with a known or putative genetic predisposition Br J Cancer 2002 87 1379 1385 10.1038/sj.bjc.6600628 12454765 PMC2376291

[B36] Slonina D Biesaga B Urbanski K Kojs Z Comparison of chromosomal radiosensitivity of normal cells with and without HRS-like response and normal tissue reactions in patients with cervix cancer Int J Radiat Biol 2008 84 421 428 10.1080/09553000802029910 18464071

[B37] Huber R Braselmann H Bauchinger M Intra- and inter-individual variation of background and radiation-induced micronucleus frequencies in human lymphocytes Int J Radiat Biol 1992 61 655 661 10.1080/09553009214551461 1349629

[B38] Bryant PE Gray L Riches AC Poppe B Messiaen L De Ridder L Vral A The G_2 _chromosomal radiosensitivity assay Int J Radiat Biol 2002 78 863 866 10.1080/09553000210144484 12428927

