# Supplementary material for: Chromosomal radiosensitivity and acute radiation side effects after radiotherapy in tumour patients - a follow-up study
Source: Radiat Oncol. 2011 Apr 7;6:32. doi: 10.1186/1748-717X-6-32 (PMC3080817; doi:10.1186/1748-717X-6-32)
Supplement: Additional file 3 — Correlation coefficients of different types of chromosome aberrations from in vitro irradiated lymphocytes. Correlation coefficients of different types of chromosome aberrations from in vitro irradiated (3 Gy) lymphocytes compared to degree of side effects and to latency of side effects in 47 patients (p-values for Spearman's rank correlation test). [file 1748-717X-6-32-S3.DOC]

Additional file 3, Table S3: Correlation coefficients of different types of chromosome aberrations from *in vitro* irradiated lymphocytes compared to degrees of side effects and to latency of side effects in 47 patients (p-values for Spearman’s rank correlation test)

| Chromosome aberration endpoint | Side effect of the skin p-value | Latency of side effects p-value |
| --- | --- | --- |
| t(Ba) | 0.81 | **0.014*** |
| t(Ab) | 0.21 | 0.18 |
| dic | 0.86 | 0.15 |
| cj | 0.44 | 0.59 |

*Bold number: significant correlation
